# Water sorption of dental resin composites: Is a new method the future?

**DOI:** 10.1111/eos.70090

**Published:** 2026-03-16

**Authors:** Lea Heckel, Renan Belli, Tabea Schüssler, Carolin Fischer, Ulrich Lohbauer

**Affiliations:** ^1^ Department of Operative Dentistry and Periodontology, Research Laboratory for Dental Biomaterials Friedrich‐Alexander‐Universität Erlangen‐Nürnberg (FAU) Erlangen Germany; ^2^ NETZSCH‐Gerätebau GmbH Selb Germany

**Keywords:** CAD/CAM, hydrolysis, ISO, Karl‐Fischer Titration, resin composite, thermogravimetry, water sorption

## Abstract

Intraoral, diffusion‐controlled water sorption leads to dimensional expansion and mechanical degradation over time. Measurement of small quantities of water in dental resin composites (RCs) is challenging, as current techniques rely on weighing approaches. Here, we evaluate Karl‐Fischer Titration (KFT) and thermogravimetry (TG) as alternatives to ISO 4049 standard. Four indirect RCs, one polymer‐infiltrated ceramic network (PICN), and one direct composite were soaked in water over 270 days, heated, and evaporated water was detected by KFT and TG. Dilatometric (DIL) and TG coupled with Fourier transform infrared spectroscopy (TG–FTIR) measurements were conducted to verify the methodology and to compare with ISO 4049 outcome. Initial water content was measured between 0.19 and 1.65 wt.%, which increased to 0.73–3.12 wt.% upon water sorption over 270 days. The direct RC performed well, indirect RCs showed significant differences, and the PICN performed comparable to indirect RCs. KFT correlated well with ISO, except for one RC. KFT proved slightly more sensitive than TG. DIL offered different linear expansion of high‐versus‐low‐absorbing materials and confirmed the water sorption rate seen by KFT. In contrast to ISO, KFT has the potential to detect initial water content allowing for a precise and kinetic quantification of the water diffusion process.

## INTRODUCTION

The interest in the effects of water on dental resin composites (RCs) peaked in the 1990s and pushed our understanding concerning sorption, elution, polymer hydrolysis, stress relaxation, and so forth rapidly forward, as much as their interdependency on monomer chemistry, polymerization, silanization, filler packing, among other aspects native to composite materials [1]. However, that interest never subsided due to the continual stream of advances in composite technology. Of late, for instance, RCs find sudden new usage as pre‐polymerized blocks for machining as indirect restorations, a sort of processing that renewed old interests regarding material interaction with water. Likewise, dental composites for 3D‐printing have reached the industrial pipeline; a clinical interest is bound to be significant—their behavior concerning interaction with water and the need for accurate and reproducible methodologies might soon foster the demand for alternative measurement approaches.

A typical “go‐to‐method” for assessing of how much water composites absorb is specified in ISO 4049 standard [2], which describes a weighing approach based on mass gain following water storage for increasingly longer periods of time. It relies on a drying procedure (vacuum storage in a desiccator at 37°C until constant mass is achieved between 24 h measurements) to set a baseline value that assumes that the sample is completely dry, thus neglecting the presence of any bound water that might be resistant against evaporation. An underestimation of the total water sorption would follow depending on how much water the material can keep bound within the polymer network through hydrogen bonding. However, in the manufacturing route, water may be added directly to the paste for better flow through extrusion channels, with that amount depending on the changing seasonal temperature and relative humidity. Another source of error contributing to uncertainties in the absorption rate and absolute values for water sorption, common to all methods based on water soaking, consists in the inability of mass changes to account for eluted monomers that may leach from the sample during storage.

Though simple and straightforward, the ISO 4049 method relies on the resolution range of lab balances (typically between 1 × 10^−4^ and 1 × 10^−5^ g) and is far from representing the state‐of‐the‐art in analytical tools for detecting water sorption in polymers. Thermogravimetry (TG), for example, makes use of highly sensitive balances (resolution up to 1 × 10^−8^ g) that can track the mass change during vaporization in an oven under controlled heating rates. Another well‐known tool to the polymer community is the Karl‐Fischer Titration (KFT) method, described in several standards such as ISO 15512 [3]. The KFT method is specific for water [4] and is used to detect and quantify very small traces in a wide range of materials [5, 6] based on a stoichiometric chemical reaction running in a closed‐system laboratorial equipment. Modern devices are able to measure very small amounts of water, especially the coulometric KFT covers a measuring range from 1 × 10^−5^ to 2 × 10^−1^ g H_2_O with a resolution of 1 × 10^−7^ g [7]. For solid samples like RCs, the analyte water has to be released by vaporization in a heating oven and transferred via an inert purge gas into the titration cell before the actual chemical conversion can take place [8]. The general chemical reaction in a methanolic system is as follows [9]:

(1) $$\begin{array}{ccc} \text{ROH} & + & {\text{SO}}_{2}+R^{\prime }N\to \left[R^{\prime }\text{NH}\right]{\text{SO}}_{3}R+H_{2}O+I_{2}+2R^{\prime }N\\ & & \to 2\left[R^{\prime }\text{NH}\right]I+\left[R^{\prime }\text{NH}\right]{\text{SO}}_{4}R \end{array}$$

where the alcohol, sulfur dioxide, and the base form an intermediate alkylsulfite salt, which is oxidized by iodine to an alkylsulfate salt under the consumption of water. Once water molecules in the material are consumed, the oxidation stops and the iodine excess marks the endpoint.

The aim was to explore both KTF and TG methods as alternative approaches to the ISO 4049 weighing method, with focus on determining the optimal temperature for water vaporization while preventing errors due to material decomposition. Finally, dilatometric (DIL) measurements are explored for in situ tracking of linear expansion curves with storage time to give insight in a physical parameter of direct clinical relevance. We tested the first null‐hypothesis of no significant differences between applied test methods and the second null‐hypothesis of no significant differences among tested materials.

## MATERIAL AND METHODS

### Materials and sample preparation

**FIGURE 1:**
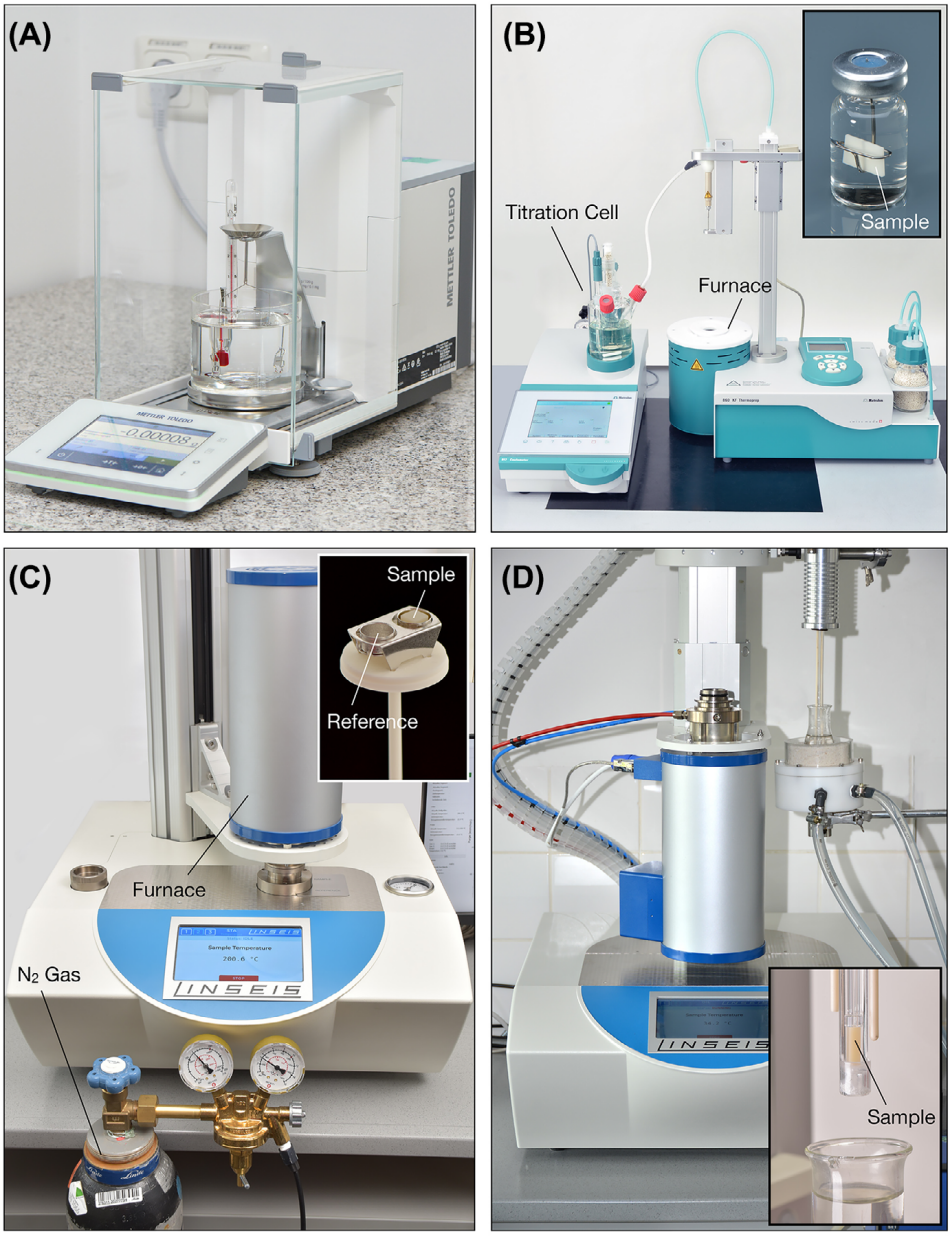
Equipment, measuring setup, and specimen geometries used for (A) high‐precision lab balance with mounted water bath for the ISO differential weighing method; (B) Karl‐Fischer Titration apparatus—inset: 10 × 10 × 1 mm^3^ specimen inside a sealed vial during water storage; (C) simultaneous thermal analysis equipment used for thermogravimetry measurements—inset: 3 × 3 × 3 mm^3^ specimen in a platinum sample holder beside an empty reference crucible; (D) vertical dilatometer—inset: 3 × 3 × 15 mm^3^ beam sample over the 37°C heated water bath.

Multiple series of measurements involving different methods and combinations of methods are performed for the materials listed in Table 1, consisting of four indirect RCs, one polymer‐infiltrated ceramic network (PICN), and one direct RC for sake of comparisons. Although the experimental design emphasized a direct evaluation between the ISO and the KFT methods, other analytical methodologies were seized upon in order to deliver additional insights: Fourier transform infrared spectroscopy coupled with TG (TG–FTIR) was used to determine the decomposition chemistry, mass loss and mass loss rate with temperature, and ultimately the optimal heating temperature for vaporization of water in heating experiments. Finally, dilatometry was explored for in situ tracking of linear expansion curves with time during water storage up to 21 days. Figure 1 shows the equipment and measurement setup used for the different methods, along with the respective specimen geometries.

**TABLE 1:** Resin composite products evaluated in this study and respective compositions.

Material	Manufacturer	Material class	Matrix composition	Filler content	Batch/shade
**Cerasmart (CS)**	GC, Japan	Indirect CAD/CAM	29 wt.% Bis‐MEPP, UDMA, DMA	71 wt.% silica, Ba‐Si‐glass fillers	2005131H A2 LT
**Lava Ultimate (LU)**	3 M, USA	Indirect CAD/CAM	20 wt.% UDMA, TEGDMA, Bis‐GMA, Bis‐EMA	80 wt.% silica 20 nm, ZrO_2_ 4–11 nm, aggregated clusters	NC60011 A2 LT
**BRILLIANT Crios (BC)**	Coltène, Switzerland	Indirect CAD/CAM	29 wt.% TEGDMA, Bis‐GMA, Bis‐EMA	71 wt.% Ba‐Si‐glass fillers <10 µm, silica < 20 nm	K12898 A2 LT
**VITA Enamic (VE)**	Vita, Germany	Indirect CAD/CAM	25 wt.% UDMA, TEGDMA	75 wt.% porous scaffold of Al‐Si‐glass	73560 4 M2 T
**Grandio Blocs (GB)**	Voco, Germany	Indirect CAD/CAM	14 wt.% UDMA, DMA	86 wt.% Ba‐Si‐glass fillers 1 µm, silica 20–40 nm	2034310 A2 LT
**Grandio SO (GS)**	Voco, Germany	Direct	11 wt.% TEGDMA, Bis‐GMA, Bis‐EMA	89 wt.% Ba‐Si‐glass fillers 1 µm, silica 20–40 nm	1740402 A2

The selection of materials in Table 1 is intended to comprise currently commercial indirect RCs exhibiting some variety in composition, specifically filler content, but also include particular microstructures, such as LU with its predominantly nanometric zirconia‐silica filler clusters and VE with its PICN architecture. The materials GB and GS were chosen due to their analog inorganic composition and silanization strategy so to approximate the comparison between an indirect and a direct composite.

To obtain specimen sizes in the dimensions required for each method (see respective sections below), block materials underwent a preparation procedure as follows: grinding the blocks (size 14) with diamond wheels (D46 down to D15 discs with final diamond grit size of 20 µm) under water cooling and subsequent sectional cutting with a low‐speed diamond saw under water irrigation, totaling approx. 25–30 min of water exposure. Specimens of the direct composite were produced in oversized Teflon molds following ISO 4049 recommendations and grinded to size as described above. After grinding, the specimens were clean‐washed with distilled water for 30 s, dried with absorbent cloth (Kimtech Science, Kimberly–Clark) for additional 30 s until apparently dry at the surface, and stored at room temperature in open vials for 24 h before further measurement of water content (re‐establishing an equilibrium between water sorption during specimen preparation with the vapor pressure of the surrounding humidity [10]; at this point referred to as “initial state”), further water storage, or further drying to constant mass. Specimens undergoing long‐term water sorption were stored at 37°C ± 1°C in an oven for different time intervals inside hermetically closed vials containing 8 mL distilled water and hanging by custom‐made wires to water‐expose all surfaces (see Figure 1B).

### Karl‐Fischer Titration (KFT)

The applied KFT approach and some material‐respective results presented in this study are based on previously published work on either indirect RC materials [11] or direct RCs [12].

A terminal specimen of size 10 × 10 × 1 mm^3^ was used throughout all KFT measurements, following pilot experiments with different dimensions. Pilot experiments were conducted to obtain the optimal ratio between specimen weight and the expected weight of water (both in dry and water‐saturated conditions) so to comply with the range of ratios recommended in ISO 15512. Assuming a water content of >1 wt.%, an experimental mass of approx. 0.2 g is recommended in ISO 15512 to keep the systematic error to a minimum. The pilot experiments were part of a former research project [10].

A first set of experiments was conducted with two materials (LU and CS) either in the “initial state” or after 14 days of water storage under a constant heating rate of 0.5°C/min up to 250°C in order to track the rate of water vaporization with temperature so to identify the optimum temperature in which only water evaporates from the specimen before any decomposition of the material takes place. Those experiments and all subsequent long‐term water‐soaked specimens were conducted in a KF coulometer (917 Coulometer, Metrohm), coupled with an oven sample processor (860 KF Thermoprep, Metrohm), in which the sample is heated to induce water vaporization, here set to 200°C, and held constant until the drift felt below a threshold of 5 µg/min. Before each experiment, the system was conditioned by removing any residual water, and a blank value was set using an empty sealed vial, after which the system's accuracy could be checked using 0.1 g of reference standard (water standard 1% Aquastar) to a 0.03 % difference. Each measurement consisted of removing the sample from its storage, wiping the surface excess water with an absorbent cloth as described above, weighing with a precision balance (Model XRS105DU, Mettler Toledo, resolution 1 × 10^−5^ g), and sealing hermetically in glass vials using aluminum septum caps and a crimping tong. During the experiment, the water vapor is carried by N_2_ gas under constant flow of 80 mL/min to the titration cell, where iodine generation according to Equation (1) was recorded under a constant voltage of 50 mV and current of 10 µA. The KF coulometer automatically calculated the percentage of water based on the given specimen weight; the values in µg/mm^3^ were converted via specimen density for comparison with the values obtained using the ISO 4049 method. KFT data were statistically treated using two‐way anova (material vs. storage time) and Student–Newman–Keuls post hoc procedure (*α* = 0.05).

### Thermogravimetry (TG)‐Coupled Fourier transform infrared spectroscopy (FTIR)

In order to evaluate the dynamic mass change while getting information on what is being released from the specimens during heating, a TG–FTIR equipment was used for the same set of samples used in the KFT method to evaluate the dynamic drift, namely, LU and CS either in the “initial state” or after 14 days of water storage. For that, approx. 25 mg of each material was weighed and heated in an open 85 µL Al_2_O_3_ crucible up to 950°C at 10°C/min in the TG equipment (TG 309 Libra, Netzsch) under constant N_2_ gas flow of 40 mL/min, which carried the vaporized water and any other decomposition gas to a pre‐heated (370°C) gas‐cell in the FTIR spectrometer (Invenio R, Bruker Optics), where its DTGS detector covers a spectral range of 4400–650 cm^−1^ with a 4 cm^−1^ resolution. Each spectrum was averaged from 16 scans and evaluated in the bruker opus software. The mass change was evaluated in the proteus software (Netzsch).

Aiming for a direct comparison to KFT, two materials (LU and GB) were selected for TGA measurements at three storage periods (1/7/21 days). For that, specimens of dimensions 3 × 3 × 3 mm^3^ were produced, stored, and dried with a cloth as described above; weighed with a precision balance (Model XRS105DU, Mettler Toledo, resolution 1 × 10^−5^ g); placed into Pt‐crucibles with lids (see inset Figure 1C); heated to 200°C at 5°C/min; and held at that temperature until a constant weight was obtained (STA PT1600, Linseis, Figure 1C), whereas under N_2_ gas flow (100 mL/min). A calibration with empty crucibles was run to obtain a correction curve for the measurements. Data were evaluated with ta software (Linseis, Version 2.3.3).

### Mass change—ISO 4049 method

Water sorption was measured on the basis of a differential weighing procedure. The four indirect RCs were prepared as described above. Disc‐shaped specimens (*n* = 5) were processed from the direct RC (GS) using a stainless‐steel mold (diameter: 15.0 ± 0.1 mm; thickness (*h*): 1.0 ± 0.1 mm). The materials were handled according to the manufacturer's instructions and cured on both sides using an LED light‐curing unit (G4, Ivoclar, 20 s, 1200 mW/cm^2^). Excess material was removed, and both surfaces were finished as described above. Specimens were then dried in a desiccator at 37 ± 1°C until a constant mass m_1_ was achieved. After drying, specimens were immersed, hanging in distilled water, and maintained at 37 ± 1°C for 7 days. After removal, they were gently blotted using an absorbent cloth and immediately weighed to obtain the wet mass *m*_2_.

Specimens were re‐dried in the desiccator at 37 ± 1°C until a second constant final dry mass m_3_ was reached.

Water sorption (*W*_sp_) was calculated in accordance with ISO 4049:

(2) $$W_{\mathrm{sp}}=\left(m_{2}-m_{3}\right)/V$$

with *W*_sp_ is the water sorption (µg/mm^3^), *m*_2_ is the mass after water immersion (µg), *m*_3_ is the final constant mass after re‐drying (µg), and *V* is the volume of the specimen (mm^3^), calculated as *V* = *r* ^2^ *πh*.

All weighing measurements were conducted under constant humidity (50%) at room temperature (23 ± 1°C) using a high‐precision balance (Model XRS105DU, Mettler Toledo, resolution 1 × 10^−5^ g). ISO 4049 was modified as measurements were taken up to 60 days water storage. ISO data were statistically compared using two‐way anova and Student–Newman–Keuls post hoc procedure (*α* = 0.05).

### Dilatometry

In order to obtain dynamic curves of linear expansion versus storage time, two materials (LU and GB) were prepared as described above (initial state) in dimensions 3 × 3 × 14 mm^3^. A vertical dilatometer (L75 PT, Linseis) was used outside the oven with the specimen chamber inserted into a water bath heated constantly to 37°C up to 21 days (see Figure 1D). Measurements were corrected by a calibration curve obtained for sapphire under the same conditions. Data were evaluated using ta software (Linseis, Version 2.3.3).

### Diffusion coefficient

As water sorption into an RC network is diffusion‐controlled, the related diffusion coefficient *D* (sorption rate) can be expressed by the following equation:

(3) $$\frac{m_{t}}{m_{\infty }}=\frac{4}{h}{\left(\frac{Dt}{\pi }\right)}^{0.5}$$

This relationship is valid for small amounts of water, as we commonly find in resin‐based composites. Based on Fick's second law, the ratio between mass of water absorbed at time *t* (*m_t_*) and mass of the saturated material (*m*_∞_) correlates to an empirical solution solely depending on time t and dimension (thickness *h* = 1 mm) [13, 14, 15]. The data from KFT measurements were taken for calculations and plotted as *m_t_*/*m*_∞_ versus *t* ^0.5^ L. The material‐specific diffusion coefficient can be derived from the initial slope of the curve and is reported in Table 2.

**TABLE 2:** Diffusion coefficients of the investigated materials based on Karl‐Fischer Titration (KFT) data (37°C, *h* = 1 mm).

Material	Diffusion coefficient (10^−8^ cm^2^/s)
Cerasmart (CS)	9.90
Lava Ultimate (LU)	14.71
BRILLIANT Crios (BC)	15.48
VITA Enamic (VE)	11.18
Grandio Blocs (GB)	10.53
Grandio SO (GS)	12.54

## RESULTS AND DISCUSSION

**FIGURE 2:**
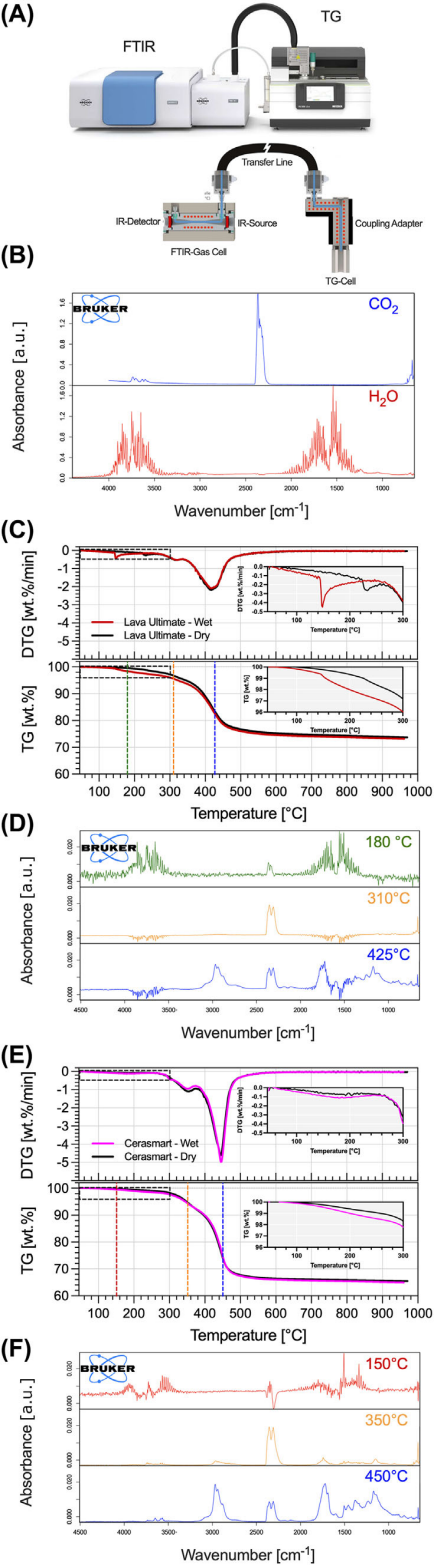
(A) Schematics of the thermogravimetry (TG)‐coupled Fourier transform infrared spectroscopy (FTIR) setup detailing the connection between both equipment, the TGA and the FTIR. (B) FTIR spectra for the calibration of CO_2_ and water. (C and E) Plots of the mass change rate (DTG in [wt.%/min]) and corresponding mass change (TG in [wt.%]) for LU and CS in dry and wet storage of 14 days. (D and F) Color‐coded FTIR spectra showing the water evaporation at 180/150°C (green plots) and additional polymer phases at 310/350°C (orange plots) and 425/450°C (blue plots). The presence of CO_2_ is observable through the temperature range. The color‐coded spectra in parts D and F correspond to the dotted lines in parts C and E and indicate the ongoing gravimetric changes over time emanating from water evaporation and later on from additional polymer decomposition.

TG–FTIR rendered mass loss profiles that can be cross‐referenced to a gaseous molecular signature up to 950°C. Figure 2C,E show the mass loss rate (DTG in [wt.%/min]) and the mass loss TG in (wt.%) for dry and wet samples of LU and CS, with FTIR excerpts from selected temperatures. Several distinct mass loss steps can be observed from the TG‐temperature curves (180°C/ 310°C/ 425°C for LU and 150°C/ 350°C/ 450°C for CS, see Figure 2D,F). At first, there is no significant change in mass up to approx. 150°C, which marks the beginning of a slight decreasing slope up to 300°C. Within that temperature range, FTIR profiles (Figure 2D,F upper row) match that of water vapor (Figure 2D lower row). The mass loss rate in that interval is distinct between CS and LU; for the latter, sudden abrupt drops take place, resulting in sharp steps in the TG slope, turning into steeper mass loss. For LU the FTIR absorbance is heightened as compared to CS. Those features suggest the presence of more free water in the material. Between ∼300°C and ∼400°C, the downward slope in mass becomes steeper even though the mass loss rate decreases; here the FTIR signature reveals the combustion leading to the release of CO_2_ (Figure 2C–F). The region between ∼400° and ∼480°C is dominated by the burning out of other carbon‐containing organic fragments, and after ∼500°C the mass stabilizes, suggesting that all organic content has been vaporized and only the inorganic filler is left. These TG–FTIR results indicate that the safe region for heating up a dental composite in order to induce sole water vaporization without other material changes is located between 150 and 250°C. The TG–FTIR experiments shown in Figure 2 were conducted on the examples of the materials LU and CS, with the assumption that the other resin‐based composites are consisting of a similar monomer composition and are behaving in a comparable manner upon temperature increase.

Literature recommendations for processing temperature exist only for pure polymethyl methacrylate (PMMA) at 180°C [16] and methacrylate‐based resins at 250°C [17]. Material properties of polymeric resins can differ substantially even if their composition is relatively similar, and thus, the temperature for water removal should be defined so that loose and bound water can be vaporized completely while avoiding the extra water that can be released by material decomposition at too‐high temperatures. In theory, the optimum is about 20–30°C lower than the decomposition temperature [18].

**FIGURE 3:**
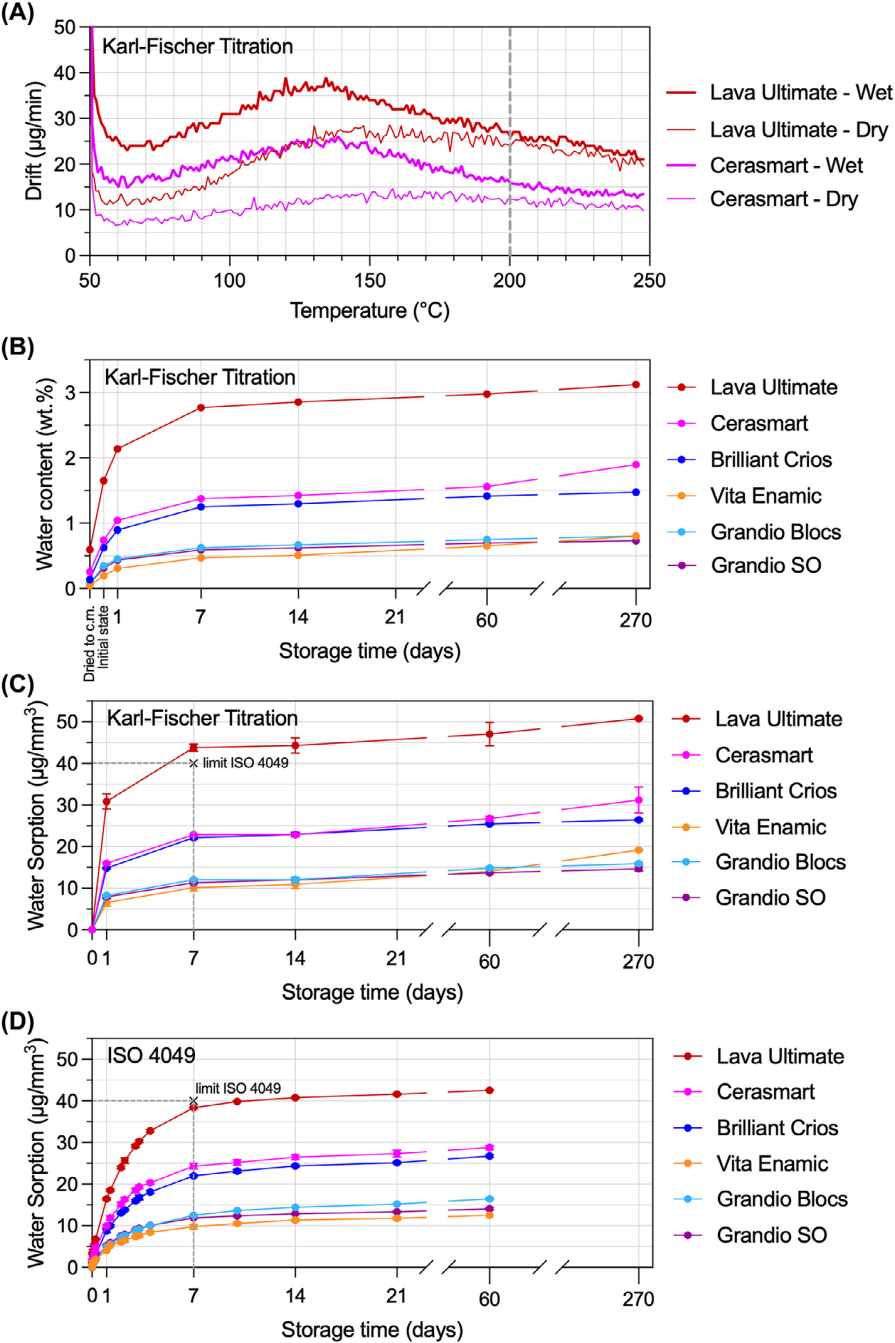
(A) Drift (water release) curves for Karl‐Fischer Titration (KFT) up to 250°C for CS and LU in dry and after 14 days of water storage showing a continuous approximation of respective (dry vs. wet) plots at elevated temperatures, representing the amount of evaporated water. (B) KFT results of water content (wt.%) up to 270 days of storage (standard deviations are smaller than symbols). (C) KFT results of water sorption (µg/mm^3^) up to 270 days of water storage (normalized to baseline). (D) Water sorption results (µg/mm^3^) from the ISO 4049 method up to 60 days. *Note*: The initial value 0 days in (C) and (D) is the value measured as the baseline (dried‐to‐constant mass).

For the purpose of complementing the TG–FTIR results, KFT experiments were conducted under continuous heating at 0.5°C/min from 50°C to 250°C in nitrogen atmosphere. The drift plot in Figure 3A shows the water release rate of dry and wet‐stored samples of CS and LU. For both wet materials the drift peak takes place at approx. 130°C, and after 200°C the dry and wet curves from both materials seem to equalize. A standard criterium to limit the titration time is the stop drift, as the sum out of the start drift (background noise) and an individually determined relative drift. ISO 15512 recommends a stop criterium lower than 5 µg/min for plastics. If 1 µg/min is chosen instead of 5 µg/min as the end point of the titration, the measurement takes 45 min longer and counts a 132 µg more of extracted water. The water content for 5 µg/min is 0.513 wt.%, and for 1 µg/min it is 0.565 wt.%, which means a variance of 0.05 wt.% water is caused by choosing different end points. This systematic error has a higher impact on dry specimens with less total water than on saturated ones. Longer measurement times do not guarantee a better accuracy because the start drift might not be constant for the whole titration time. It can fluctuate from 3 to 7 µg/min despite long conditioning times before starting KFT. At the end of long titration times, the background noise of the system is often lower due to a reduced humidity in the closed system. The initial start drift does not fit anymore, and results are calculated too low. A stop drift of 5 µg/min was evaluated as appropriate for RCs [10].

The question, if “all” water in the specimen is recorded, remains unanswered. Formulating a precise definition of “all” water—on the surface, sterically, or chemically bonded—that shall be detected is not clearly possible. Like many resin‐based, plastic materials, dental composites cannot be dried completely [19]. Varying the temperature and sample size led to different percentual results, but the repeatability was excellent for the 10 × 10 × 1.0 mm^3^ geometry (in contrast to 3 × 3 × 3 mm^3^ cubes for instance). Thin samples of 1 mm thickness are common in experiments on diffusion processes [20]. The specimen size can also influence the relation between weight and water content, which need to stay in an optimal range for both water‐saturated and dried‐to‐constant‐mass specimens. Once coulometric KFT is only appropriate for an expected water release of 10 µg to 200 mg water, one vial should contain 500–5000 µg water [21], a requisite also met well for the 100 mm^3^ plates (3403 µg water when stored for 7 days and 955 µg water when dried 7 days in desiccator). KFT is specific for water, but drawing conclusions on the binding state remains difficult, even if KFT advantageously provides extra kinetic information with the aid of the drift curve. Nevertheless, the direct chemical conversion of water is beneficial for such small amounts in comparison to the weighing procedure described in ISO 4049.

### Methods comparison

Although the various methods proved comparable water sorption kinetics, the first null‐hypothesis of this study had to be rejected as KFT was sensitive to initially stored water in the polymer structure.

After direct water storage of the samples, excessive surface water needed to be removed before KFT measurements. ISO 4049 describes a method to dry specimens with a cloth before weighing, even though this conceals a considerable uncertainty. In order to validate this procedure, CS samples were stored in water for 5 min, dried with a cloth, and titrated. This resulted in slightly higher water amounts, namely, 0.722 ± 0.001 wt.% in comparison to CS samples in their initial state (0.610 ± 0.005 wt.%). The total water content was higher, but the error is systematic and well‐predictable. Hence, the ISO recommended procedure for removal of attached surface water is assumed to be similarly applicable for KFT.

In Figure 3B, the absolute water content for the KFT method is plotted for the increasing storage times, with the water sorption plotted in Figure 3C. Direct comparisons between KFT and the ISO 4049 method can be done by comparing Figure 3C,D. It becomes evident that for all materials apart from LU, the results from single time periods as well as the entire trend of water sorption are comparable between KFT and the ISO 4049 method. What the ISO 4049 cannot assess is the amount of water that remains in the material even if a procedure for obtaining a baseline (such as the dried‐to‐constant‐mass procedure) is undertaken; a drier baseline than that cannot be obtained only by drying in a desiccator. The KFT method can measure the water in the material that resists this drying procedure. In LU, this makes a difference (about 0.6 wt.%), because the absorbed water (plot in Figure 3D) is translated into µg/mm^3^ from the total mass gained (plot in Figure 3C). The vertical shift toward higher water sorption values for LU surpasses the limit recommended by ISO 4049 at 7 days (<40 µg/mm^3^). One can argue that the water that remains in a material after the drying‐to‐constant‐mass procedure is not actually absorbed during storage, but so is not the water that is “lost” between the initial state and the drying procedure. Regarding practical benefits, the water determination with KFT is as time‐consuming as the ISO 4049, but the specimen is lost at every measurement period with the KFT method. However, if KFT is regarded singularly and the reference point for water sorption could be chosen not at constant mass but in the initial air‐dry stadium, the method is easier and specific for water.

**FIGURE 4:**
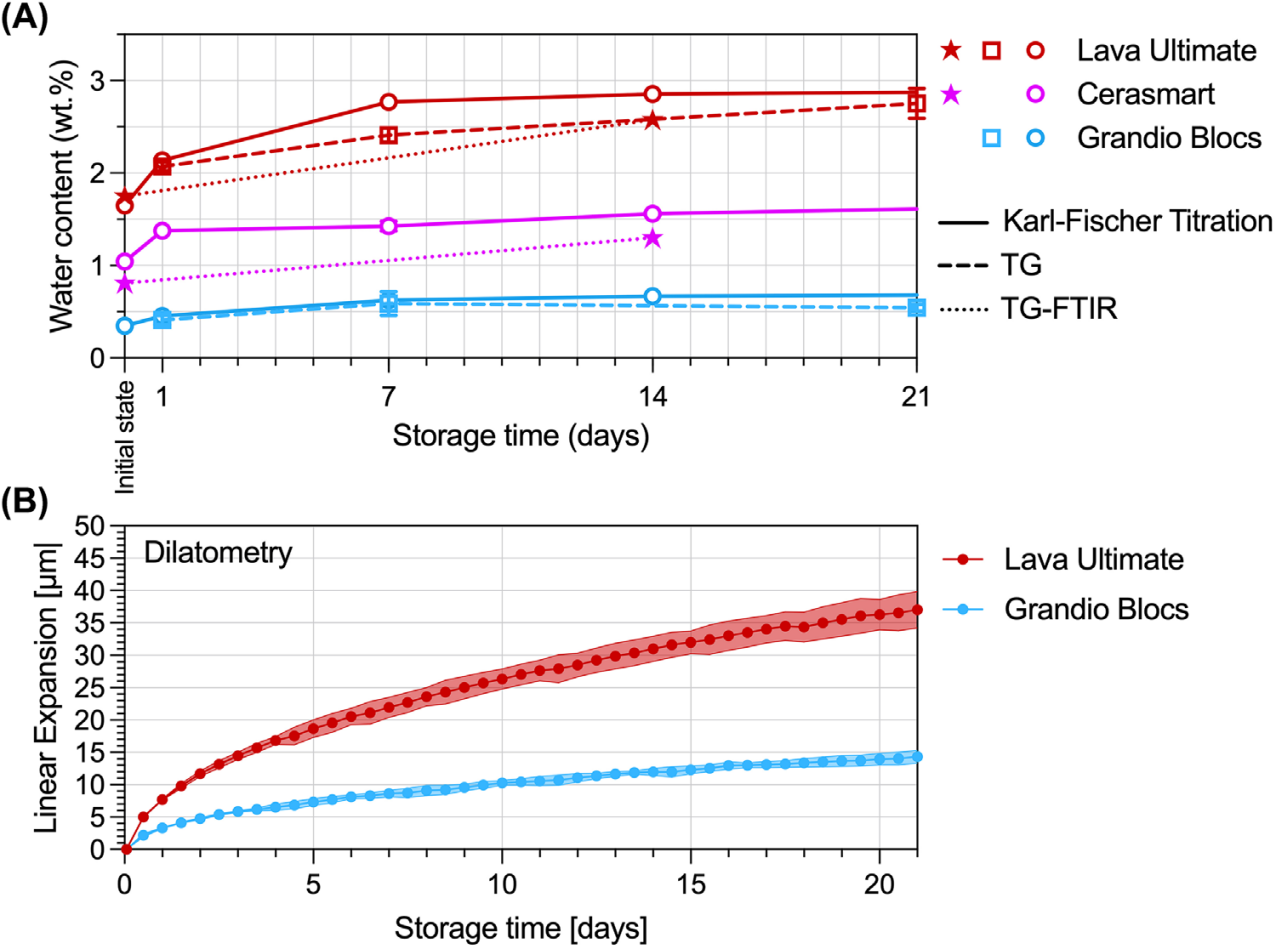
(A) Comparison of water content (wt.%) according to Karl‐Fischer Titration (KFT), thermogravimetry (TG), and TG–Fourier transform infrared spectroscopy (FTIR) methods for LU, CS, and GB at different storage intervals (note: dotted lines connect the two TG–FTIR measurements and do not indicate a certain behavior). (B) In situ dilatometry in water at 37°C body temperature depicting the linear expansion of LU and GB up to 21 days (results are plotted in µm (related to a 14 mm long test specimen); means and standard deviations of *n* = 3 measurements). A comparison between both measurements underlines the kinetics of water diffusion and the qualitative correlation of mass gain by water sorption with the linear expansion of both materials.

Figure 4A compares the values obtained by KFT with those using TGA with or without FTIR support (TG–FTIR). Although single values vary slightly, with KFT being seemingly more sensitive, the general trend is that both methods are comparable. A certain limitation of the TGA measurements without coupled FTIR analysis is that the measured mass loss cannot be truly assigned to evaporated water but could further overlap at elevated temperatures with the onset of material decomposition. In Figure 4B, we complement the TGA/TG–FTIR/KFT evaluations by making in situ measurements with a dilatometer for a high‐water‐absorbing material (LU) versus a low‐absorbing material (GB). The difference between them in terms of evolution of expansion with time fits well with the water sorption kinetics measured by KFT as well as by ISO 4049 method. The underlying question was if mass gain by water sorption translates into either viscoelastic relaxation, internal stress build‐up, or in hydrolytic expansion of resin‐based composites. Diffusion kinetics are a wide field of research and have only been touched here, but further reading is provided elsewhere [22]. Dilatometry here shows in a qualitative manner that linear expansion of resin‐based composite materials follows the diffusion kinetics of water sorption. The remaining study materials were not involved in the DIL testing, but based on the ISO and KFT results, one can assume that their linear expansion follows water sorption and hence stays between the two extreme materials LU and GB. Specimen dimensions for dilatometry are different to KFT, especially in terms of thickness (prolonged diffusion depth), and so they still do not fully saturate after 21 days and keep expanding.

Comparing the specimen dimensions used in KFT (plate‐shaped specimens [10 × 10 × 1 mm^3^]), TGA (cubic specimens [3 × 3 × 3 mm^3^]), and dilatometry (bar‐shaped specimens [3 × 3 × 14 mm^3^]) with ISO dimensions (disc‐shaped specimens [*d* = 15 mm, *h* = 1 mm]), it becomes obvious that a direct comparison is biased as more bulky specimens offer retarded diffusion kinetics. Even by using normalized data (in wt.% or µg/mm^3^), an uncertainty remains, regarding time‐wise comparisons. For that reason, the measurements were conducted in a continuous manner until complete (>95%) water saturation was reached.

### Material comparisonssca

The second null‐hypothesis was rejected, as significant differences in water sorption were observed among the tested materials.

Two‐way anova showed that both “material” (*p* < 0.001) and “storage time” (*p* < 0.001) affected the water content of specimens measured in wt.% (KFT data) and in µg/mm^2^ (ISO data). Interaction between the both factors was also significant (*p* < 0.001), regardless of the analyzed data set.

**FIGURE 5:**
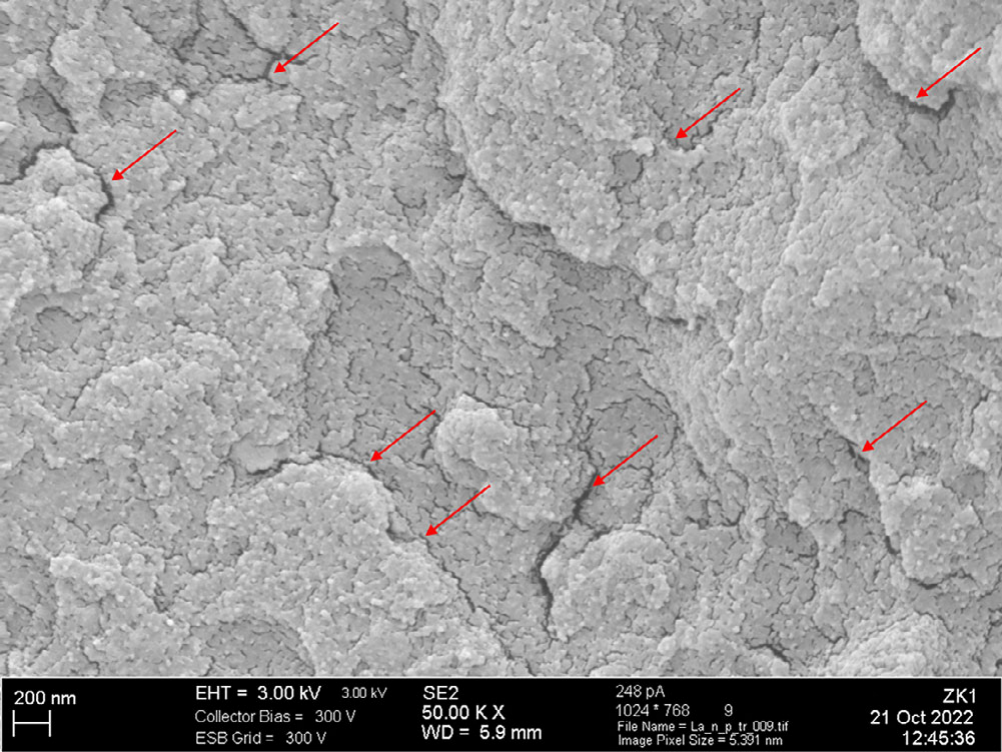
High‐resolution scanning electron microscopic image (SEM) of the LU internal microstructure after water storage and dehydration, taken from a virgin fracture surface. Arrows indicate microcrack formation, most likely responsible for the significantly highest water sorption of the materials under investigation.

Experimentally, some inferences between specific material features and their affinity to water can be drawn from our results. The CAD/CAM composite LU stands out exhibiting the significantly highest water absorption and fastest absorption rate. Based on clinical findings, LU proved an abnormal debonding after short service times, culminating in the retraction by the manufacturer of its clinical indication for single crowns [23, 24]. The problem might lay in dimensional changes caused by an increased affinity to water, as demonstrated by dilatometry. In inlays, material expansion leads to cuspal deflection, but in crowns, material expansion results in the increase in lumen dimensions, pulling it orthogonally from the bonded interface. An experimental study by Schepke *et al.* proved the dimensional changes for LU after water storage and concluded on the evolution of interfacial stress, leading to terminal debonding and fracture of the crowns [25, 26, 27]. The composition of LU is very unique, containing clusters of zirconia nanoparticles and further silica nanoparticles. Additionally, some materials, in particular LU and BC, showed an increasing number of microcracks after longer storage times in water. Figure 5 shows a high‐resolution image of a fractured LU surface, exhibiting small microcracks and voids, serving as reservoir for water. Evidence for microcrack formation in LU is also documented in literature [28]. It can be speculated that such crack formation might contribute to elevated water sorption and material expansion. In that context, internal and interconnecting defects are also shown for the material VE [29], but in this case, it does not translate into elevated water sorption and dimensional expansion of the material. A strong, reinforcing effect is expected from the interpenetrating network microstructure of the polymer and the ceramic phases.

The water sorption kinetics of CS and BC are fairly identical, exhibiting no statistically significant difference comparing KFT data points up to 60 days. The polymer fraction in both materials is quite similar (29 wt.%). In consequence, the water sorption after 270 days of storage did not differ much (CS: 1.9 wt.%, BC: 1.5 wt.%). Further, GB and GS, a CAD/CAM resin block and a direct composite, both produced by the same manufacturer, behave nearly identically (no statistical significant difference comparing KFT data points up to 270 days). This is presumably due to their very similar filler content (which is inversely correlated to water sorption), filler silanization procedures, and polymer fraction. Their similarity is nevertheless remarkable because GS has to be polymerized chairside and the microstructure is probably more inhomogeneous. GB, in contrast, is polymerized under pressure at higher temperatures in a standardized lab setting, suggesting an improved polymerization sequence and overall material performance. However, GS absorbed the least amount of water during the observation time. A study with experimental composites pointed out that the conversation rate of polymers was not crucial for the total amount of absorbed water, which is in according to our findings herein [30]. It can be speculated that elevated water sorption might correlate with mechanical degradation of a material in a long‐term perspective. An earlier study investigated on this hypothesis and found a correlation between hydrolytic and mechanical degradation for the materials under investigation [31]. Therein, GB and GS outperformed the materials CS and BC, but especially LU, which offered a significant drop in fracture toughness and biaxial strength after 60 days of water storage.

**FIGURE 6:**
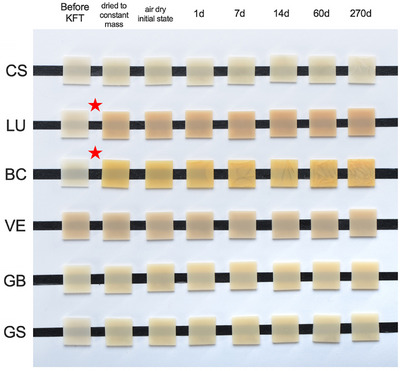
Optical photograph of specimens measured in Karl‐Fischer Titration (KFT) (at 200°C) after different drying and storage periods. Color changes are visible, especially for LU and BC after the drying to constant mass procedure (asterisks).

As a further, descriptive outcome of the study, we observed distinct color changes of the material upon heating in the KFT oven up to 200°C. Figure 6 shows a qualitative comparison of the transition from initial tooth shades A2 into a stained appearance, especially for the materials BC and LU, already after constant mass drying and KFT measurement at 200°C. Color changes in materials BC and LU might indicate the onset of polymer decomposition, although TG–FTIR could not detect any decomposition products at 180°C (compare Figure 2D for material LU). This implies that the fit of the process temperature is crucial to the KFT measurement, and a certain trade‐off between the precision of the measured water release and the systematic errors created by the onset of polymer decomposition has to be taken into account.

The significant difference in water sorption kinetics between high‐ (LU) and low‐ (GB) water‐absorbing materials is of prominent clinical relevance. As from the beginning of market introduction of the class of indirect resin‐based CAD/CAM chairside blocks, the target clinical indication was the single tooth full crown. With time, the clinical reality proved a high incidence of crown delamination or high bulk fracture rates, resulting in market retraction of the full crown indication (which, in turn, highly limits the clinical indication range for this material class). As discussed above, own clinical findings investigated on clinical failure reasons and established a correlation between high‐water sorption of indirect resin‐based composites and crown delamination (debonding) as a clinical failure criterion [25, 26, 27].

## CONCLUSIONS

KFT can be assessed to be a valid complement in dental materials science. It is a unique method, specific for water detection, and is already a reference method in related fields and part of some general norms. The KFT method has been shown to be efficient, precise, reproducible, and easy to use and might assist in answering questions concerning hydrolysis and degradation of dental materials. A comparison to literature according to ISO 4049 was possible respecting the necessary processing steps. The resin‐based composites under investigation showed significant differences in either initial water content (only detectable by KFT) or water sorption over time (shown by ISO 4049 and KFT). The materials GB and GS showed the least water sorption compared to CS and BC, but all materials outperformed the material LU, which did not even fulfill the ISO 4049 requirements for polymer‐based restorative materials.

## AUTHOR CONTRIBUTIONS

**Writing—original draft**: Lea Heckel, Renan Belli. **Investigation**: Lea Heckel, Tabea Schüssler, Carolin Fischer. **Methodology**: Lea Heckel, Renan Belli, Tabea Schüssler, Carolin Fischer, Ulrich Lohbauer. **Writing—review and editing**: Renan Belli, Carolin Fischer, Ulrich Lohbauer. **Formal analysis**: Renan Belli, Carolin Fischer, Ulrich Lohbauer. **Project administration**: Renan Belli, Ulrich Lohbauer. **Conceptualization**: Carolin Fischer, Ulrich Lohbauer. **Supervision**: Ulrich Lohbauer. **Funding acquisition**: Ulrich Lohbauer. **Resources**: Ulrich Lohbauer.

## CONFLICT OF INTEREST STATEMENT

The authors declare no conflicts of interest.

## FUNDING INFORMATION

The study was not financed by external sources.
